# Supplementary material for: Associations between in vitro, in vivo and in silico cell classes in mouse primary visual cortex
Source: Nat Commun. 2023 Apr 24;14:2344. doi: 10.1038/s41467-023-37844-8 (PMC10126114; doi:10.1038/s41467-023-37844-8)
Supplement: Supplementary file 1 — Supplementary Information [file 41467_2023_37844_MOESM1_ESM.pdf]

Associations between *in vitro*, *in vivo* and *in silico* cell classes in mouse primary visual cortex

Yina Wei[1,2,*], Anirban Nandi[2], Xiaoxuan Jia[2], Joshua H. Siegle[2], Daniel Denman[3], Soo Yeun Lee[2], Anatoly Buchin[2,4], Werner Van Geit[5], Clayton P. Mosher[6], Shawn Olsen[2], Costas A. Anastassiou[6-9, 10,*]

1. Zhejiang Lab, Hangzhou 311100, China
2. Allen Institute for Brain Science, Seattle, WA 98109, USA
3. University of Denver, Denver, CO 80208, USA
4. Cajal Neuroscience Inc, Seattle, WA 98102, USA
5. Blue Brain Project, École Polytechnique Fédérale de Lausanne (EPFL) Campus Biotech, Geneva 1202, Switzerland
6. Department of Neurosurgery, Cedars-Sinai Medical Center, Los Angeles, CA 90048, USA
7. Department of Neurology, Cedars-Sinai Medical Center, Los Angeles, CA 90048, USA
8. Board of Governors Regenerative Medicine Institute, Cedars-Sinai Medical Center, Los Angeles, CA 90048, USA
9. Center for Neural Science and Medicine, Department of Biomedical Sciences, Cedars-Sinai Medical Center, Los Angeles, CA 90048, USA
10. Lead contact

*Corresponding author: CAA (costas.anastassiou@cshs.org)
and YW (weiyina039@zhejianglab.com)

SUPPLEMENTARY INFORMATION

Figure S1

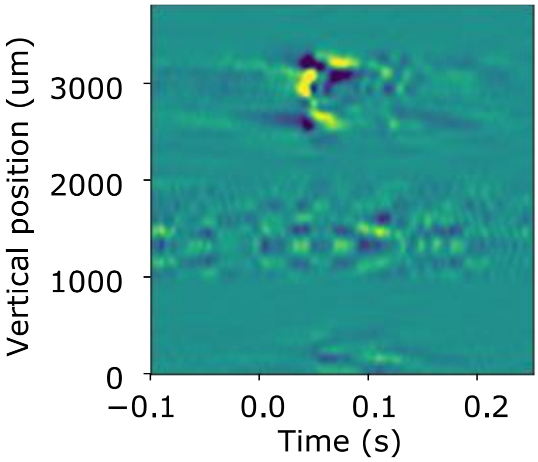

Figure S1. Visual stimulus (flashes) evoked a strong response evident in the current source density (CSD) analysis. X axis is the time relative to stimulus onset (0: time of stimulus onset). Y axis is the vertical position of the Neuropixels probe (associated with the cortical depth). The sink of CSD (yellow) first occurred at the vertical position of 2740 um. This position is ascribed as the middle of layer 4 and denoted as depth 0.

Figure S2

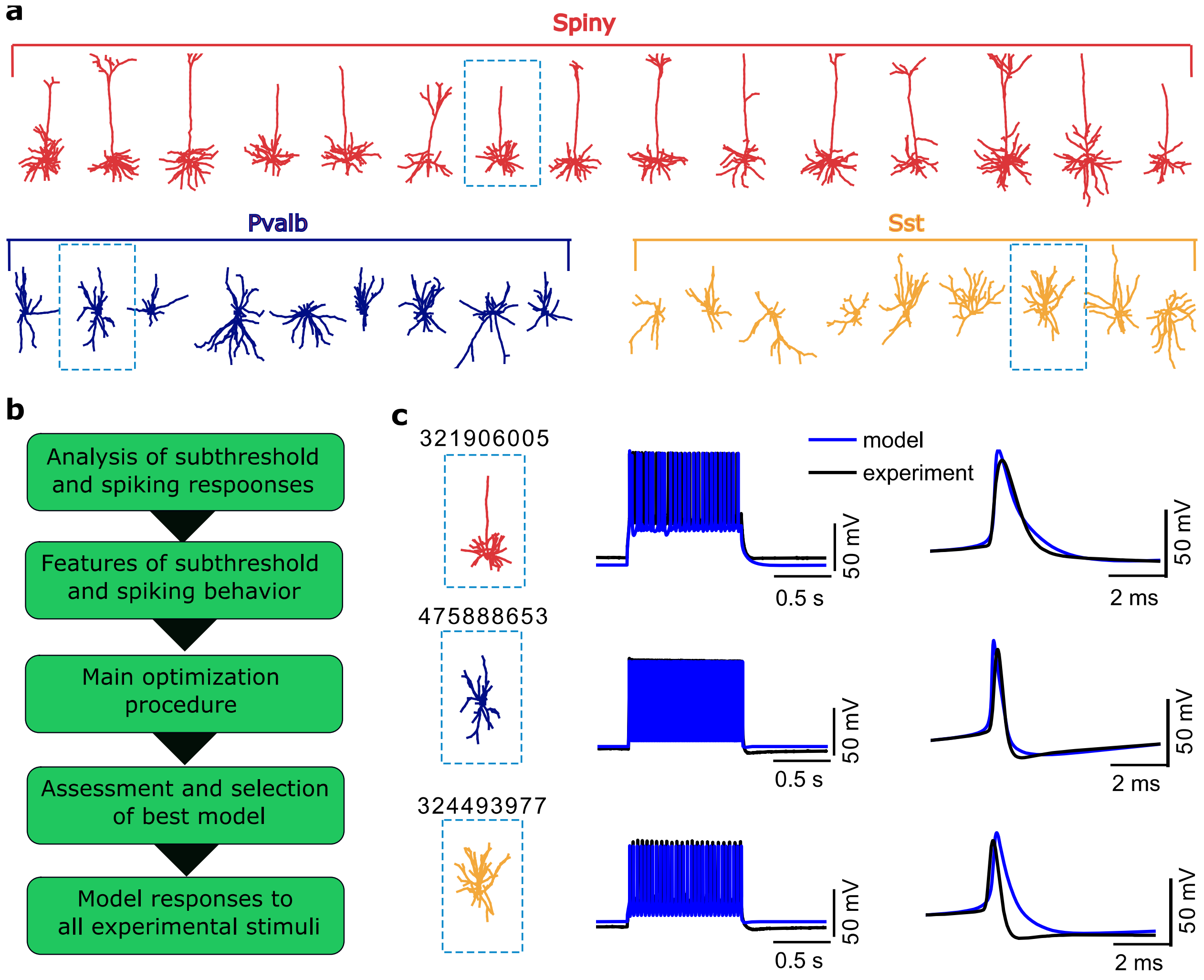

Figure S2. Computational optimization workflow for the generation of single-cell ("all-active") models of mouse cortical neurons. a) The morphology of spiny (red), and aspiny (blue: Pvalb; orange: Sst) neurons is reconstructed from imaged biocytin fills performed in whole-cell patch-clamp experiments. **b)** Sequence of steps comprising the model generation and optimization workflow. **c)** Three example cell models (top: a spiny cell; middle: a Pvalb cell; bottom: a Sst cell). Left column: reconstructed neuron morphology (box indicates the morphology in panel **a**); Middle column: simulated dynamics for the individual models resulting from the optimization workflow (intracellular stimulus: 1s somatic current injection; black: experimental trace; blue: model trace). Right column: the somatic action potential waveform. For more information on the computational optimization procedure see Methods.

Figure S3

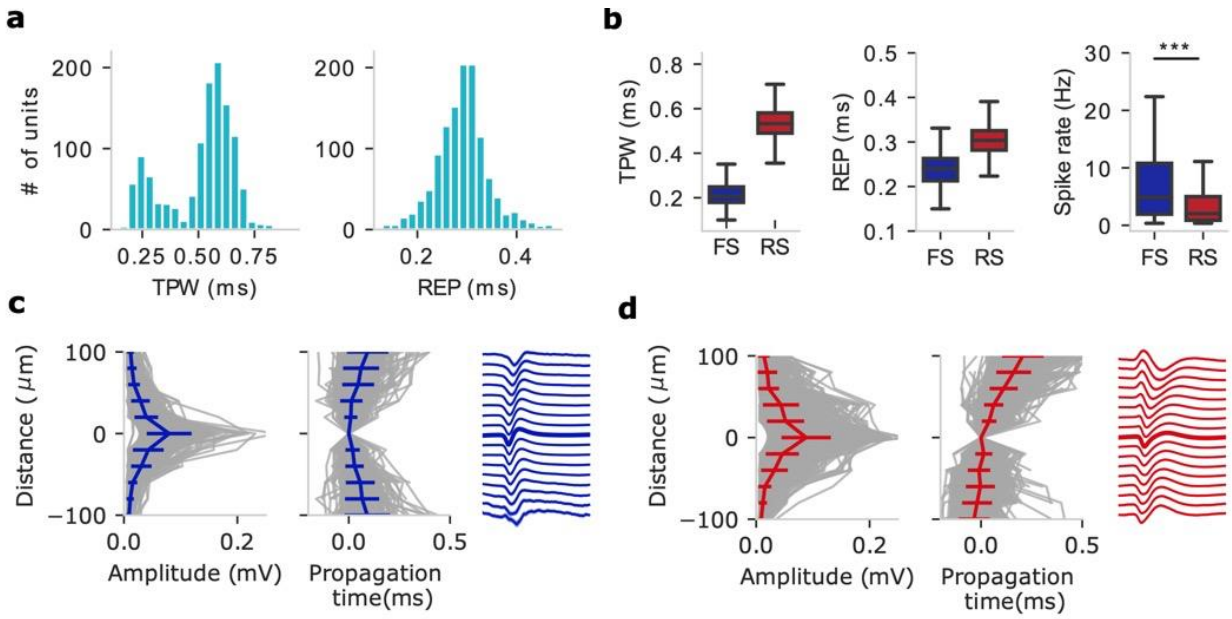

Figure S3. **One- and multi-channel extracellular action potential (EAP) feature analysis of one-channel clusters RS and FS. a**) The distribution of one-channel EAP features across all recorded units: trough-peak width (TPW) and repolarization time (REP). **b**) Comparison of the spike rate shows that the narrow TPW cluster constitutes a fast-spiking (FS) group while the wide TPW cluster is attributed to a regular-spiking (RS) group. Box plots show center line as median, box limits as upper (75%) and lower (25%) quartiles. The whiskers extend from the box limits by 1x the interquartile range. n=281 FS units, n=923 RS units. Mann-Whitney U test, two-sided, $p=3.55*10^{-18}$, ***$p<0.001$. **c-d**) EAP amplitude and propagation time (calculated as a multi-channel feature) of FS (**c,** n=281) and RS (**d**, n=923) as function of the cortical depth. Gray lines indicate individual units while colored lines indicate mean ± SD (standard deviation). Note the asymmetry in propagation time of EAPs between FS (**c**, blue) and RS (**d**, red).

Figure S4

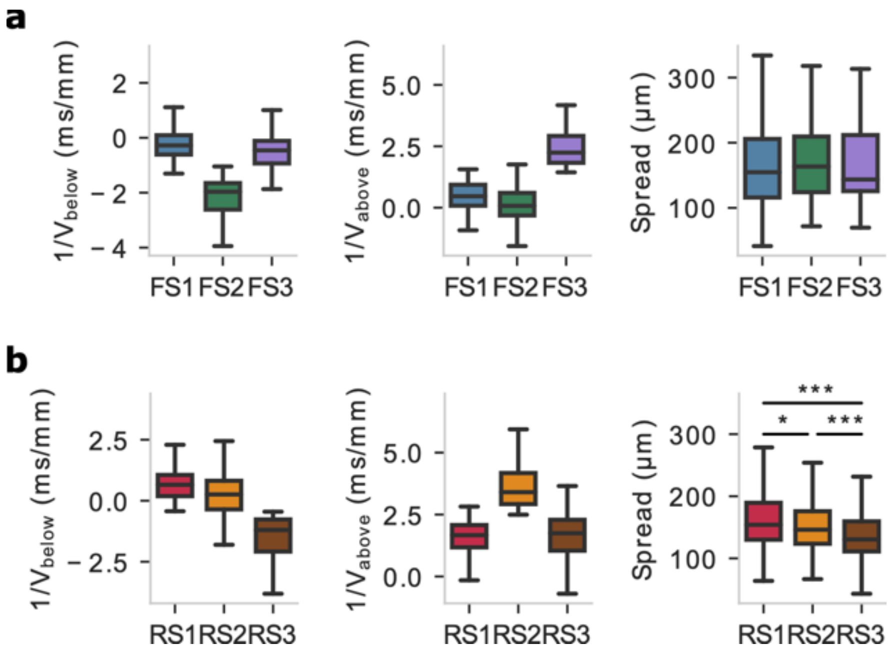

Figure S4. **Multi-channel extracellular action potential (EAP) feature analysis of multi-channel clusters RS1-3 and FS1-3.** Comparisons and boxplots of multi-channel extracellular action potential (EAP) features among (**a**) fasting-spiking (FS) clusters: FS1 (n=130), FS2 (n=82), FS3 (n=69), and (**b**) fasting-spiking (FS) clusters: RS1 (n=479), RS2 (n=235), RS3 (n=209) . Box plots show center line as median, box limits as upper and lower quartiles. The notches represent confidence intervals around the median. The whiskers extend from the box limits by 1x the interquartile range. Comparison of spread among FS1-3, Kruskal-Wallis H-test, F=0.457, p=0.795, p-values corrected using the Holm-Bonferroni method for multiple tests; Comparison of spread among RS1-3, Kruskal-Wallis H-test, F=48.2, $p = 3.34*10^{-11}$, p-values corrected using the Holm-Bonferroni method for multiple tests, *p<0.05, ***p<0.001.

Figure S5

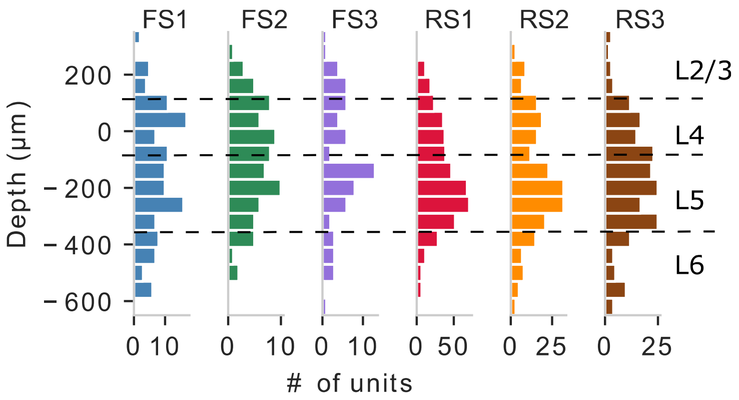

Figure S5. **Distribution of extracellular action potential (EAP) multi-channel waveform clusters along the cortical depth.** Distribution of EAP waveform clusters (FS1-3 and RS1-3) in mouse primary visual cortex (V1) as function of cortical depth relative to layer 4 (depth 0 indicates the center of layer 4). Localization of layer 4 center is shown in **Fig. S1**.

Figure S6

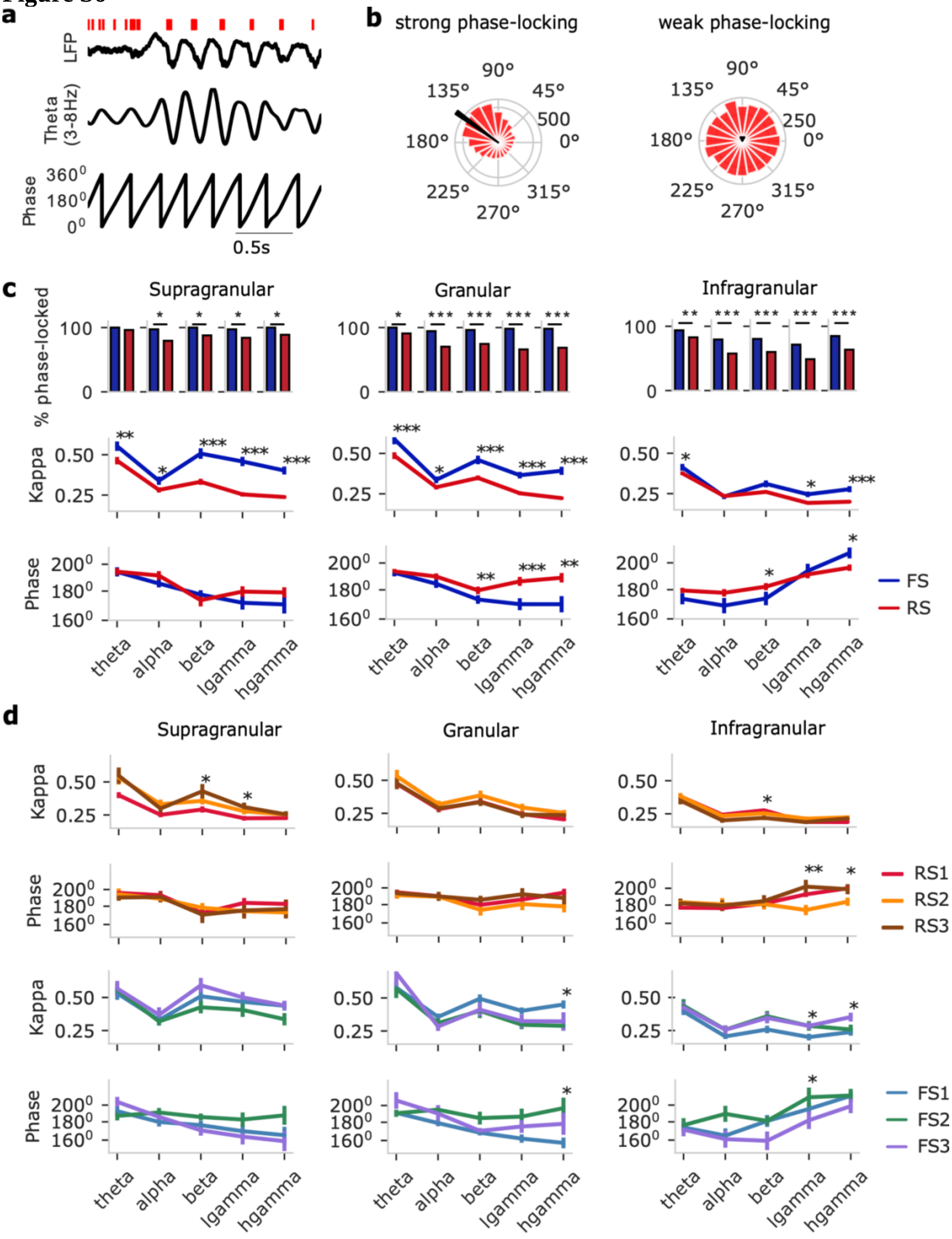

Figure S6. **Spike-phase relationships of one- and multi-channel clusters. a**) Example of raw local field potential (LFP, top, black trace) and the raster plot of a spiking unit recorded simultaneously (top, red), the filtered LFP at theta band (middle) and the instantaneous phase computed via the Hilbert transform (bottom). 180^0 (or π) represent the trough of each cycle. **b**) Examples of the phase distribution of a strongly phase-locked unit (left), and a weakly entrained unit (right). The direction of the black arrow represents the mean preferred phase,

while the length of the black arrow represents the kappa. **c**) Phase locking across layers. Top: The percentage of phase-locked units in fast-spiking (FS, blue) vs. regular-spiking (RS, red) at different LFP frequency bands: theta, alpha, beta, low gamma (lgamma), high gamma (hgamma). Supragranular layer: *n*=37 FS, *n*=107 RS; Granular layer: *n* = 54 FS, *n*=185 RS; Infragranular layer: *n*=112 FS, *n*=453 RS. Two sample z test for proportions with p-values corrected by the Holm-Bonferroni method for multiple tests. *p<0.05, **p<0.01, ***p<0.001. Bottom: kappa and preferred phase. Data are presented as mean ± SEM (standard error of mean) . Mann-Whitney U test, two-sided, *p<0.05, **p<0.01, ***p<0.001. **d**) Kappa and preferred phase of RS1-3 and FS1-3 across layers. Top: RS1 (red), RS2 (orange), and RS3 (brown); Bottom: FS1 (blue), FS2 (green), FS3 (purple). Data are presented as mean ± SEM. Mann-Whitney U test, two-sided, *p<0.05, **p<0.01.

Figure S7

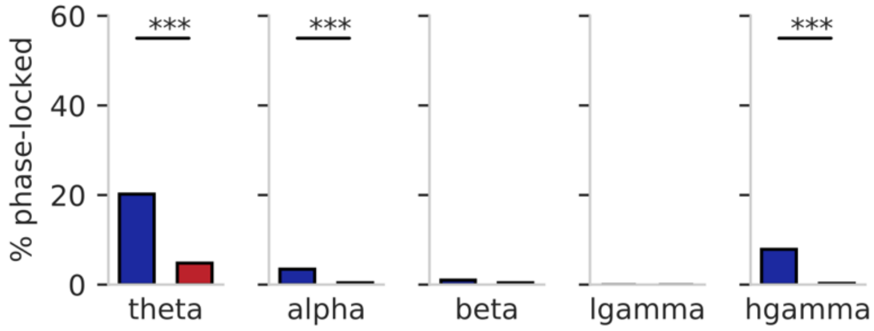

Figure S7. **Spike-phase relationship on one-channel clusters during spontaneous activity.** The percentage of phase-locked units during spontaneous activity for FS (fast-spiking, blue, n=203) and RS (regular-spiking, red, n=750) clusters at different LFP (local field potential) frequency bands: theta, alpha, beta, low gamma (lgamma), high gamma (hgamma). Two sample z test for proportions with p values corrected by the Holm-Bonferroni method for multiple tests. ***p<0.001. The total number of units activated during spontaneous activity are *n*=1705 but we only included the units activated during both spontaneous activity and drifting gratings (*n*=953) to show the input- and behavior-dependent properties.

Figure S8

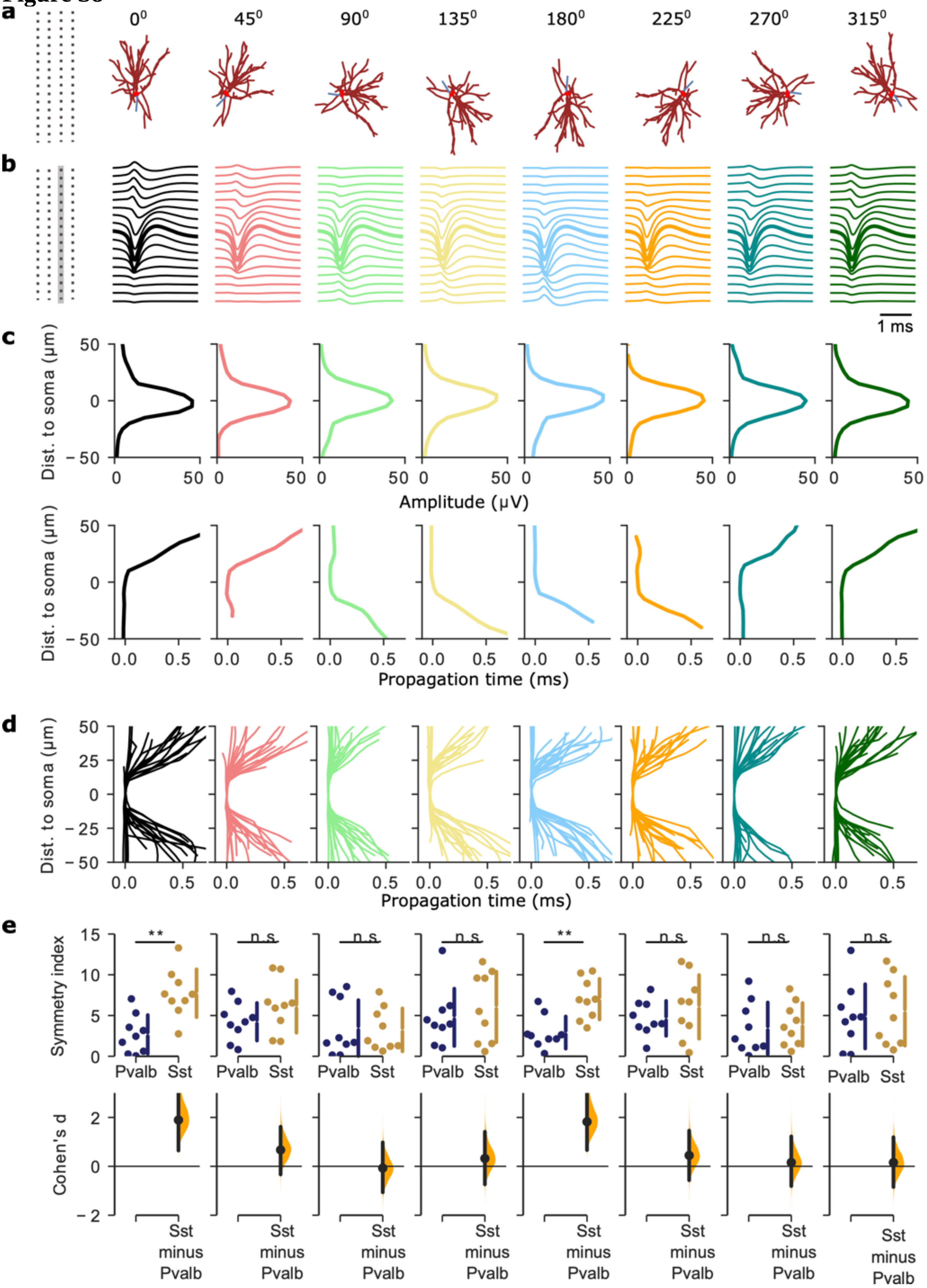

Figure S8. **The effect of the angle between cellular morphology and the simulated Neuropixels probe traversing along the cortical depth axis**. **a-c**) Example of the rotation effect of the cellular morphology on the extracellular action potential (EAP) features of one Sst cell (cell ID: 324493977). **b**) The multi-channel EAPs correspond to the rotation angles (shown

in panel **a**). **c**) EAP features can change as a function of distance-to-soma at different rotation angles. The rotation effect is shown on two EAP features: amplitude (top) and propagation time (bottom). While the EAP amplitude remains broadly unchanged with rotation, the EAP propagation time changes considerably with rotation angle. **d-e**) The rotation effect on the EAP features of Pvalb and Sst is shown across cellular models (Pvalb: n = 9; Sst: n = 9; see also **Table S1**). **d**) The EAP propagation time is shown as a function of distance to soma for all aspiny models at different rotation angles (reported in panel **a**, top). Each line corresponds to one cell. **e)** Symmetry index of the EAP propagation changes with rotation angle with certain rotation angles exacerbating differences in symmetry index between Pvalb vs. Sst while others leave them statistically undetected (dark blue: Pvalb, n=9; orange: Sst, n=9). Top: symmetry index between Pvalb and Sst for various rotation angles (from left to right, as shown in panel **a**) and models. Each dot represents one model. Lines designate mean ± standard deviation (SD). Bottom: Cohen's d shows the effect size and direction. $^{**}p<0.01$, n.s. represents no significance.

Table S1. Metadata about origin of experimental *in vitro* data used to generate single-cell models. *In vitro* electrophysiology data and the reconstructed morphology are generated for each whole-cell patched neuron from mouse primary visual cortex. Data for each experiment available online (https://celltypes.brain-map.org).

	Cell ID	Cre lines	Layer	Dendrite type
1	395830185	Scnn1a	L4	spiny
2	476218657	Scnn1a	L4	spiny
3	320207387	Scnn1a	L4	spiny
4	422738880	Nr5a1	L4	spiny
5	476457450	Nr5a1	L4	spiny
6	386049446	Nr5a1	L4	spiny
7	321906005	Nr5a1	L4	spiny
8	485931158	Rorb	L4	spiny
9	479179020	Rorb	L4	spiny
10	478586295	Rorb	L5	spiny
11	468120757	Scnn1a	L5	spiny
12	484559000	Rorb	L5	spiny
13	476048909	Rorb	L5	spiny
14	314822529	Rorb	L5	spiny
15	318808427	Nr5a1	L2/3	spiny
16	487667205	Pvalb	L4	aspiny
17	475888653	Pvalb	L4	aspiny
18	471085676	Pvalb	L5	aspiny
19	469801569	Pvalb	L5	aspiny
20	469610831	Pvalb	L5	aspiny
21	396608557	Pvalb	L5	aspiny
22	333604946	Pvalb	L2/3	aspiny
23	330080937	Pvalb	L5	aspiny
24	318331342	Pvalb	L4	aspiny
25	485058595	Sst	L5	aspiny
26	482690728	Sst	L5	aspiny
27	478396248	Sst	L5	aspiny
28	466664172	Sst	L5	aspiny
29	485466109	Sst	L2/3	aspiny
30	473540161	Sst	L2/3	aspiny
31	324493977	Sst	L2/3	aspiny
32	476104386	Sst	L6	aspiny
33	313862306	Sst	L6	aspiny
