# Supplementary material for: Associations between in vitro, in vivo and in silico cell classes in mouse primary visual cortex
Source: Nat Commun. 2023 Apr 24;14:2344. doi: 10.1038/s41467-023-37844-8 (PMC10126114; doi:10.1038/s41467-023-37844-8)
Supplement: Supplementary file 2 — Reporting Summary [file 41467_2023_37844_MOESM2_ESM.pdf]

Corresponding author(s): Costas A. Anastassiou , Yina Wei

Last updated by author(s): Mar 8, 2023

Reporting Summary

Nature Portfolio wishes to improve the reproducibility of the work that we publish. This form provides structure for consistency and transparency in reporting. For further information on Nature Portfolio policies, see our Editorial Policies and the Editorial Policy Checklist.

Statistics

For all statistical analyses, confirm that the following items are present in the figure legend, table legend, main text, or Methods section.

n/a	Confirmed	
☐	☒	The exact sample size (*n*) for each experimental group/condition, given as a discrete number and unit of measurement
☐	☒	A statement on whether measurements were taken from distinct samples or whether the same sample was measured repeatedly
☐	☒	The statistical test(s) used AND whether they are one- or two-sided<br>*Only common tests should be described solely by name; describe more complex techniques in the Methods section.*
☐	☒	A description of all covariates tested
☐	☒	A description of any assumptions or corrections, such as tests of normality and adjustment for multiple comparisons
☐	☒	A full description of the statistical parameters including central tendency (e.g. means) or other basic estimates (e.g. regression coefficient) AND variation (e.g. standard deviation) or associated estimates of uncertainty (e.g. confidence intervals)
☐	☒	For null hypothesis testing, the test statistic (e.g. *F*, *t*, *r*) with confidence intervals, effect sizes, degrees of freedom and *P* value noted<br>*Give P values as exact values whenever suitable.*
☒	☐	For Bayesian analysis, information on the choice of priors and Markov chain Monte Carlo settings
☐	☒	For hierarchical and complex designs, identification of the appropriate level for tests and full reporting of outcomes
☐	☒	Estimates of effect sizes (e.g. Cohen's *d*, Pearson's *r*), indicating how they were calculated

Our web collection on statistics for biologists contains articles on many of the points above.

Software and code

Policy information about availability of computer code

Data collection	https://allensdk.readthedocs.io/en/latest/visual_coding_neuropixels.html<br>https://celltypes.brain-map.org<br>https://allensdk.readthedocs.io/en/latest/_static/examples/nb/ecephys_optotagging.html
Data analysis	https://github.com/yinawei/Mouse_V1_EAP_Analysis<br>https://github.com/yinawei/Mouse-all-active-models-EAP<br>10.5281/zenodo.7679748<br>10.5281/zenodo.7679762

For manuscripts utilizing custom algorithms or software that are central to the research but not yet described in published literature, software must be made available to editors and reviewers. We strongly encourage code deposition in a community repository (e.g. GitHub). See the Nature Portfolio guidelines for submitting code & software for further information.

Data

Policy information about availability of data

All manuscripts must include a data availability statement. This statement should provide the following information, where applicable:

- Accession codes, unique identifiers, or web links for publicly available datasets
- A description of any restrictions on data availability
- For clinical datasets or third party data, please ensure that the statement adheres to our policy

The in vivo Neuropixels dataset is available for download in Neurodata Without Borders (NWB) format via the AllenSDK23: https://allensdk.readthedocs.io/en/latest/visual_coding_neuropixels.html

The Neurodata Without Borders files are also available on the DANDI Archive23: https://gui.dandiarchive.org/#/dandiset/000021

The in vitro electrophysiology data and the reconstructed morphology used to generate single-cell models are available in: https://celltypes.brain-map.org
The cell ID used in the paper was listed in the Table S1.

The optotagging experimental data set with Pvalb and Sst neurons is available through: https://allensdk.readthedocs.io/en/latest/_static/examples/nb/ecephys_optotagging.html

Source data are provided with this paper.

Human research participants

Policy information about studies involving human research participants and Sex and Gender in Research.

Reporting on sex and gender	NA
Population characteristics	NA
Recruitment	NA
Ethics oversight	NA

Note that full information on the approval of the study protocol must also be provided in the manuscript.

Field-specific reporting

Please select the one below that is the best fit for your research. If you are not sure, read the appropriate sections before making your selection.

☒ Life sciences ☐ Behavioural & social sciences ☐ Ecological, evolutionary & environmental sciences

For a reference copy of the document with all sections, see nature.com/documents/nr-reporting-summary-flat.pdf

Life sciences study design

All studies must disclose on these points even when the disclosure is negative.

Sample size	All in vivo recordings come from the Allen Brain Observatory Visual Coding Neuropixels dataset23, accessible via the AllenSDK (https://allensdk.readthedocs.io/en/latest/visual_coding_neuropixels.html) and the DANDI Archive (https://gui.dandiarchive.org/#/dandiset/000021). Recordings were performed in awake, head-fixed mice allowed to run freely on a rotating disk. During the recording, mice either passively viewed visual stimuli (flashes) or viewed a mean-luminance gray screen. Data were collected from 25 wild-type C57BJ/6J mice (24 male, 1 female), and 8 Pvalb-IRES-Cre (6 male, 2 female) and 12 Sst-IRES-Cre (8 male, 4 female) crossed with an Ai32 channelrhodopsin reporter line79. Cre+ cells from Ai32 lines are highly photosensitive, due to the expression of Channelrhodopsin-280. The Neuropixels probe can record from 384 contacts across 3.84 mm of issue coverage (selectable from 960 available sites on a 10 mm length shank). In this study, we analyzed recordings from the primary visual cortex (V1). All extracellular spike data were acquired with Neuropixels probes21, with 30 kHz sampling rate (which achieves 0.033 ms temporal resolution) and a 500 Hz analog high-pass filter. Spike times and waveforms were automatically extracted from the raw data using KiloSort2.
Data exclusions	No data was excluded
Replication	All replication strategies were successful.
Randomization	Animals were fully randomized and all data were included in the analyses (Figure 2). Feature definition and all analyses are detailed in the Methods section.

Blinding	Investigators were blinded to group allocation.

Reporting for specific materials, systems and methods

We require information from authors about some types of materials, experimental systems and methods used in many studies. Here, indicate whether each material, system or method listed is relevant to your study. If you are not sure if a list item applies to your research, read the appropriate section before selecting a response.

Materials & experimental systems

n/a	Involved in the study	
☒	☐	Antibodies
☒	☐	Eukaryotic cell lines
☒	☐	Palaeontology and archaeology
☐	☒	Animals and other organisms
☒	☐	Clinical data
☒	☐	Dual use research of concern

Methods

n/a	Involved in the study	
☒	☐	ChIP-seq
☒	☐	Flow cytometry
☒	☐	MRI-based neuroimaging

Animals and other research organisms

Policy information about studies involving animals; ARRIVE guidelines recommended for reporting animal research, and Sex and Gender in Research

Laboratory animals	All in vivo recordings come from the Allen Brain Observatory Visual Coding Neuropixels dataset, accessible via the AllenSDK (https://allensdk.readthedocs.io/en/latest/visual_coding_neuropixels.html) and the DANDI Archive (https://gui.dandiarchive.org/#/dandiset/000021). Recordings were performed in awake, head-fixed mice allowed to run freely on a rotating disk. During the recording, mice either passively viewed visual stimuli (flashes) or viewed a mean-luminance gray screen. Data were collected from 25 wild-type C57BJ/6J mice (24 male, 1 female), and 8 Pvalb-IRES-Cre (6 male, 2 female) and 12 Sst-IRES-Cre (8 male, 4 female) crossed with an Ai32 channelrhodopsin reporter line79. Cre+ cells from Ai32 lines are highly photosensitive, due to the expression of Channelrhodopsin-280. The Neuropixels probe can record from 384 contacts across 3.84 mm of issue coverage (selectable from 960 available sites on a 10 mm length shank). In this study, we analyzed recordings from the primary visual cortex (V1). All extracellular spike data were acquired with Neuropixels probes21, with 30 kHz sampling rate (which achieves 0.033 ms temporal resolution) and a 500 Hz analog high-pass filter. Spike times and waveforms were automatically extracted from the raw data using KiloSort2.
Wild animals	NA
Reporting on sex	Data were collected from 25 wild-type C57BJ/6J mice (24 male, 1 female), and 8 Pvalb-IRES-Cre (6 male, 2 female) and 12 Sst-IRES-Cre (8 male, 4 female) crossed with an Ai32 channelrhodopsin reporter line79. Cre+ cells from Ai32 lines are highly photosensitive, due to the expression of Channelrhodopsin-2.
Field-collected samples	NA
Ethics oversight	All experiments adhered to institutional guidelines (IACUC protocol 2104)

Note that full information on the approval of the study protocol must also be provided in the manuscript.
